# The trimeric solution structure and fucose-binding mechanism of the core fucosylation-specific lectin PhoSL

**DOI:** 10.1038/s41598-018-25630-2

**Published:** 2018-05-17

**Authors:** Kazuhiko Yamasaki, Tomoko Yamasaki, Hiroaki Tateno

**Affiliations:** 1 Biomedical Research Institute, National Institute of Advanced Industrial Science and Technology (AIST), Tsukuba, 305-8566 Japan; 2 Biotechnology Research Institute for Drug Discovery, National Institute of Advanced Industrial Science and Technology (AIST), Tsukuba, 305-8568 Japan

## Abstract

The core α1–6 fucosylation-specific lectin from a mushroom *Pholiota squarrosa* (PhoSL) is a potential tool for precise diagnosis of cancers. This lectin consists of only 40 amino acids and can be chemically synthesized. We showed here that a synthesized PhoSL peptide formed a trimer by gel filtration and chemical cross-linking assays, and determined a structure of the PhoSL trimer by NMR. The structure possesses a β-prism motif with a three-fold rotational symmetry, where three antiparallel β-sheets are tightly connected by swapping of β-strands. A triad of Trp residues comprises the structural core, forming NH–π electrostatic interactions among the indole rings. NMR analysis with an excess amount of fucose revealed the structural basis for the molecular recognition. Namely, fucose deeply enters a pocket formed at a junction of β-sheet edges, with the methyl group placed at the bottom. It forms a number of hydrophobic and hydrogen-bonding interactions with PhoSL residues. In spite of partial similarities to the structures of other functionally related lectins, the arrangement of the antiparallel β-sheets in the PhoSL trimer is novel as a structural scaffold, and thus defines a novel type of lectin structure.

## Introduction

Fucosylation is a common modification in glycoproteins, and is classified into several types with regard to the modes of linkage, i.e., α1–2, α1–3, α1–4, and α1–6. Among them, the α1–6 fucosylation is typically found at the first *N*-acetyl-D-glucosamine (GlcNAc) of the *N*-glycan core (called “core” fucosylation) in the mammalian systems, and is keenly related to cancer development^1^. Most prominently, the level of core fucosylation of *N*-glycan in alpha-fetoprotein (AFP) increases in hepatocellular carcinomas (HCC) and carcinoma metastatic to the liver, but not in benign liver diseases, such as acute viral hepatitis, chronic hepatitis, or liver cirrhosis^2^. This was explained by selective secretion of fucosylated glycoproteins produced in hepatocyte through bile duct, although such bile duct does not exist in tissues of the carcinomas^1^. Therefore, lectins specific to core fucosylation should be particularly useful for the diagnostics of these cancers.

A lectin highly specific to core α1–6 fucosylation was recently isolated from a mushroom *Pholiota squarrosa* (PhoSL)^3^. PhoSL binds only to core-fucosylated glycans, but not to the other types of fucosylated glycans, as revealed by frontal affinity chromatography^4^. In addition, this lectin showed stronger affinity than that of a clinically used lectin *Lens culinaris* agglutinin (LCA). Therefore, researchers started to use PhoSL for detecting core fucosylation in diagnosis of cancers and related diseases, i.e., colorectal, prostate, pancreatic, and liver cancers as well as a chronic pancreatitis that is a background of pancreatic ductal adenocarcinoma^5–9^.

The PhoSL molecule purified from the mushroom consists of only 40 residues and can therefore be chemically synthesized^3^. It is likely that PhoSL exists in a trimeric or tetrameric form, as shown by gel filtration, mass spectrometry, and gel electrophoresis analyses^3^. This lectin showed an extreme stability against heat treatment, e.g., 100 °C for 30 min, and also against incubation at pH 2–11. PhoSL is therefore suitable for its use as a medical tool in that it is easy to produce and to keep in the active form. Moreover, it would be easier to incorporate unnatural amino acids by means of the chemical synthesis, in order to produce lectins with novel specificities.

In the present study, we determined a novel structure of the PhoSL trimer by NMR, and elucidated interaction with fucose. The results provided a basis for understanding the structural stability and recognition of fucosylated glycans.

## Results and Discussion

### Trimerization of the PhoSL peptide

**Figure 1:**
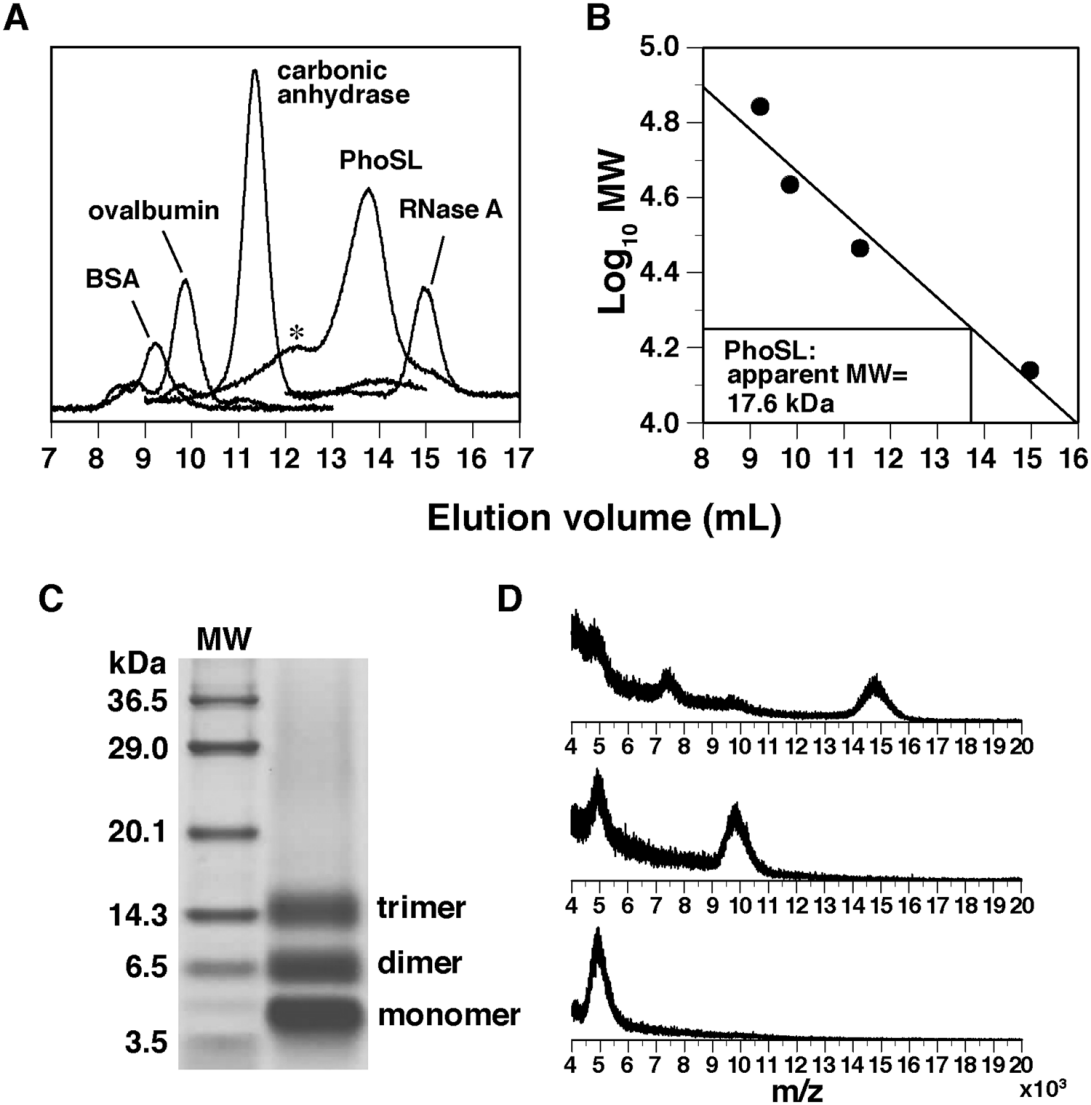
Trimeric state of the PhoSL peptide as revealed by gel filtration and chemical cross-linking analyses. Shown are (**A**) elution profile and (**B**) relation between elution volume and molecular mass of the proteins in gel filtration analysis, (**C**) SDS-PAGE gel after the cross-linking reaction with EDC and NHS, and (**D**) mass spectra of the three respective bands in (C). Asterisk in (A) shows the peak for the presumable hexamer of PhoSL.

While the PhoSL molecule purified from the mushroom has been suggested to form a trimer or a tetramer^3^, we determined the oligomeric state of the chemically synthesized peptide. By gel filtration analysis with marker proteins, the molecular weight of PhoSL was estimated to be 17.6 kDa (Fig. 1A,B). This value is slightly less than four times the size of the PhoSL monomer (4.5 kDa).

Next, a cross-linking reaction was carried out by using 1-ethyl-3-(3-dimethylaminopropyl)carbodiimide (EDC) and *N*-hydroxysuccinimide (NHS). These reagents covalently link amino groups in Lys residues or at the N-terminus and carboxyl groups in Asp/Glu residues or at the C-terminus, when pairs of the linkable groups are located in close proximity. After the reaction, three bands were observed in sodium dodecyl sulfate (SDS)-polyacrylamide gel electrophoresis (PAGE) (Fig. 1C). These corresponded to monomer, dimer, and trimer, respectively, as determined by mass spectrometry (Fig. 1D). The monomer and dimer are likely to be observed after incomplete reaction or linking of pairs within the same polypeptide that blocks the inter-chain linking. In contrast, the trimer band should represent the oligomeric state of PhoSL, because a band for the tetramer is not observed at all. In this cross-linking approach, presumable dimerization of this trimer, i.e., hexamerization, cannot be excluded; the linkable pairs may not exist around the interface between the two trimers. In the gel filtration profile, we indeed observed a minor peak for a presumable hexamer (~27 kDa), although it is now clear that the main peak is for the trimer (Fig. 1A). Thus, the oligomeric state of the PhoSL peptide was determined by combination of the two methods.

### Solution structure of PhoSL trimer

We used the chemically synthesized peptide without isotope labeling for the NMR analyses. In the preliminary analyses, the protein dissolved in phosphate buffer (pH 6.0), which showed significant precipitation (data not shown). Instead, the protein solution was clear when dissolved in Tris buffer (pH 7.5). Therefore, we used this buffer for all the NMR experiments in the present study.

**Figure 2:**
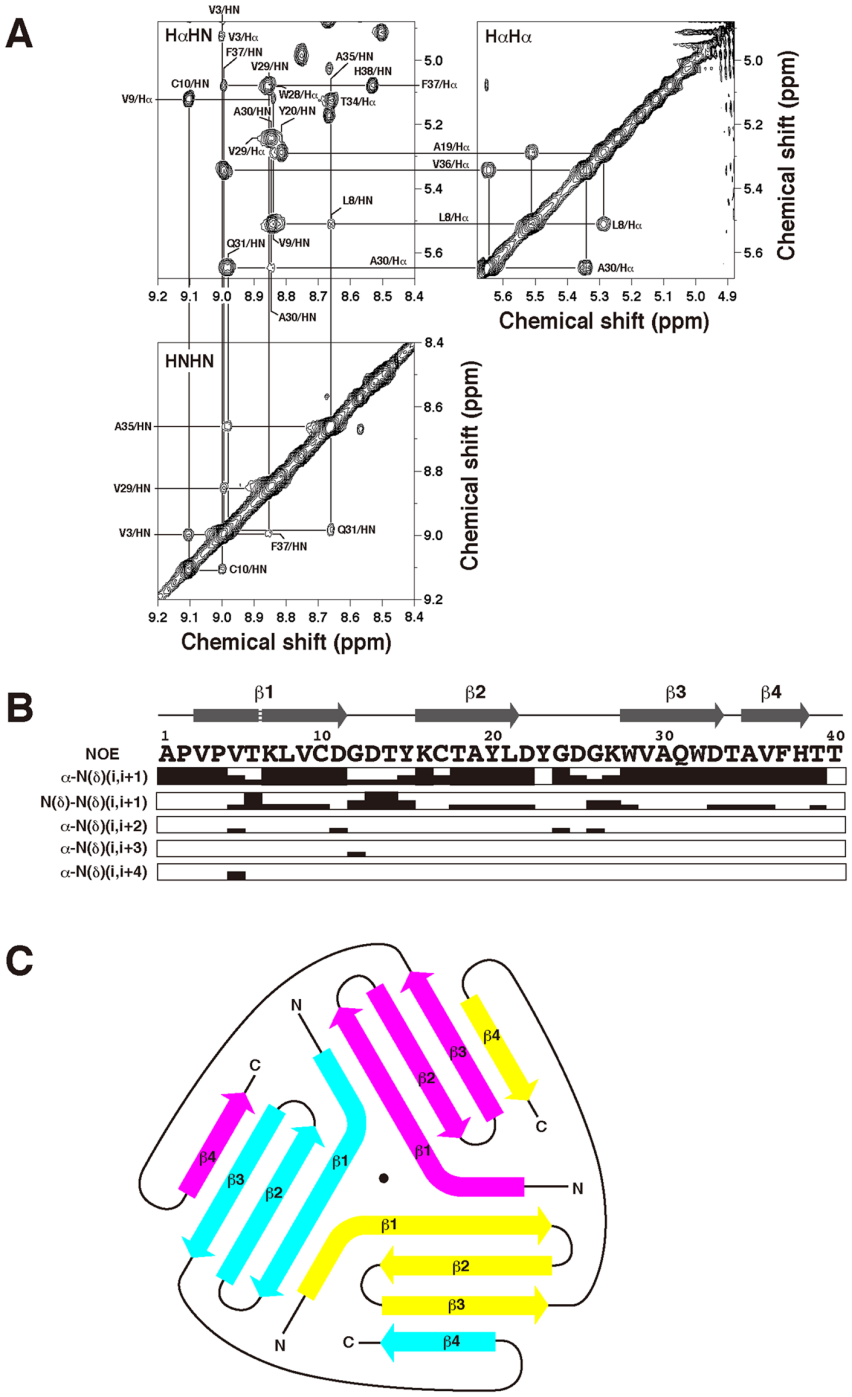
Secondary structure of PhoSL. (**A**) Tiles of a NOESY spectrum (100 ms mixing time) showing intensive sequential H_α_H_N_ NOEs, which are characteristics of β-strands, and H_α_H_α_ and H_N_H_N_ NOEs, which reveal connection of β-strands in β-sheets. The spectrum was recorded at 308 K and frequency of 900 MHz. (**B**) Profile of sequential and medium-range NOEs. Heights of the bars distinguish the large, medium, and small intensities, corresponding to distance constraints of ≤3.5 Å, ≤4.5 Å, and ≤6.0 Å, respectively. Regions of the β-strands appearing in the structural ensemble, as shown by Procheck-NMR^29^, are indicated above the sequence. A kink between Thr6 and Lys7 in β-strand 1 is shown by a white broken line. (**C**) A schematic representation of arrangements of the strands in β-sheets. The three monomers are distinguished by different colors. The dot indicates the center of rotational symmetry.

The NMR spectra of PhoSL contained a single set of resonances, which implies a structural symmetry of the PhoSL trimer. Backbone Nuclear Overhauser effect (NOE) connections revealed that PhoSL possessed a secondary structure rich in β-strands (Fig. 2A,B). Namely, we observed intense sequential H_α_H_N_ NOEs, implying close proximity between these protons, which are characteristic of the extended peptide conformation of β-strands^10^. In contrast, we did not observe intense sequential H_N_H_N_ NOEs and medium-range H_α_H_N_ NOEs (|i–j| = 3 or 4), which are characteristics of helices^10^. The backbone H_α_H_α_ NOEs and H_N_H_N_ NOEs were observed between the β-strands, revealing the arrangements of the strands in β-sheets (Fig. 2A).

Within the β-sheets, however, we cannot distinguish between intermolecular and intramolecular NOE connections of the β-strands, due to the symmetry. Therefore, in the structure calculation, ambiguities with regard to this distinction were introduced (see Materials and Methods section; Table 1). Following these trials in calculations, the connections between β-strands were determined as those shown in Fig. 2C. Therefore in the final calculation, a part of NOEs in the interfaces between the strands were fixed with regard to intermolecular or intramolecular ones.

**Table 1:** Structural statistics for the NMR structure of PhoSL trimer.

Structural constraints (per trimer)		
Sequential NOE constraints^a^	606	
Intramolecular		606
Medium-range NOE constraints (2 ≤ |i–j| ≤ 4)^a^	378	
Intermolecular		45
Ambiguous		333
Long-range NOE constraints (|i–j| > 4)^a^	1107	
Intramolecular		567
Intermolecular		321
Ambiguous		219
Hydrogen bond constraints	120	
Torsion angle constraints	81	
χ^1^		75
χ^2^		6
Total	2292	
**Characteristics**		
	Ensemble of 20 structures	Minimized mean structure
RMSD from constraints		
NOEs (Å)	0.0019 ± 0.0008	0.001
Torsion angles (degrees)	0.000 ± 0.000	0
Noncrystallographic symmetry (kcal/mol)	0.00 ± 0.00	0.003
Van der Waals energy (kcal/mol)^b^	6.5 ± 1.0	5.7
RMSD from the ideal geometry		
Bond lengths (Å)	0.0008 ± 0.00004	0.0008
Bond angles (degrees)	0.227 ± 0.003	0.224
Improper angles (degrees)	0.157 ± 0.004	0.153
Average RMSD from the unminimized mean structure (Å)		
Backbone N, C_α_, and C′	0.35 ± 0.06	
All nonproton atoms	0.68 ± 0.09	
Ramachandran plot		
Most favored region (%)	87.6	90.9
Additionally allowed region (%)	12.4	9.1
Generously allowed region (%)	0	0
Disallowed region (%)	0	0

^a^Each NOE corresponds to three constraints for the symmetric trimer.

^b^Values calculated with the repulsive nonbonded energy function in the CNS software package.

**Figure 3:**
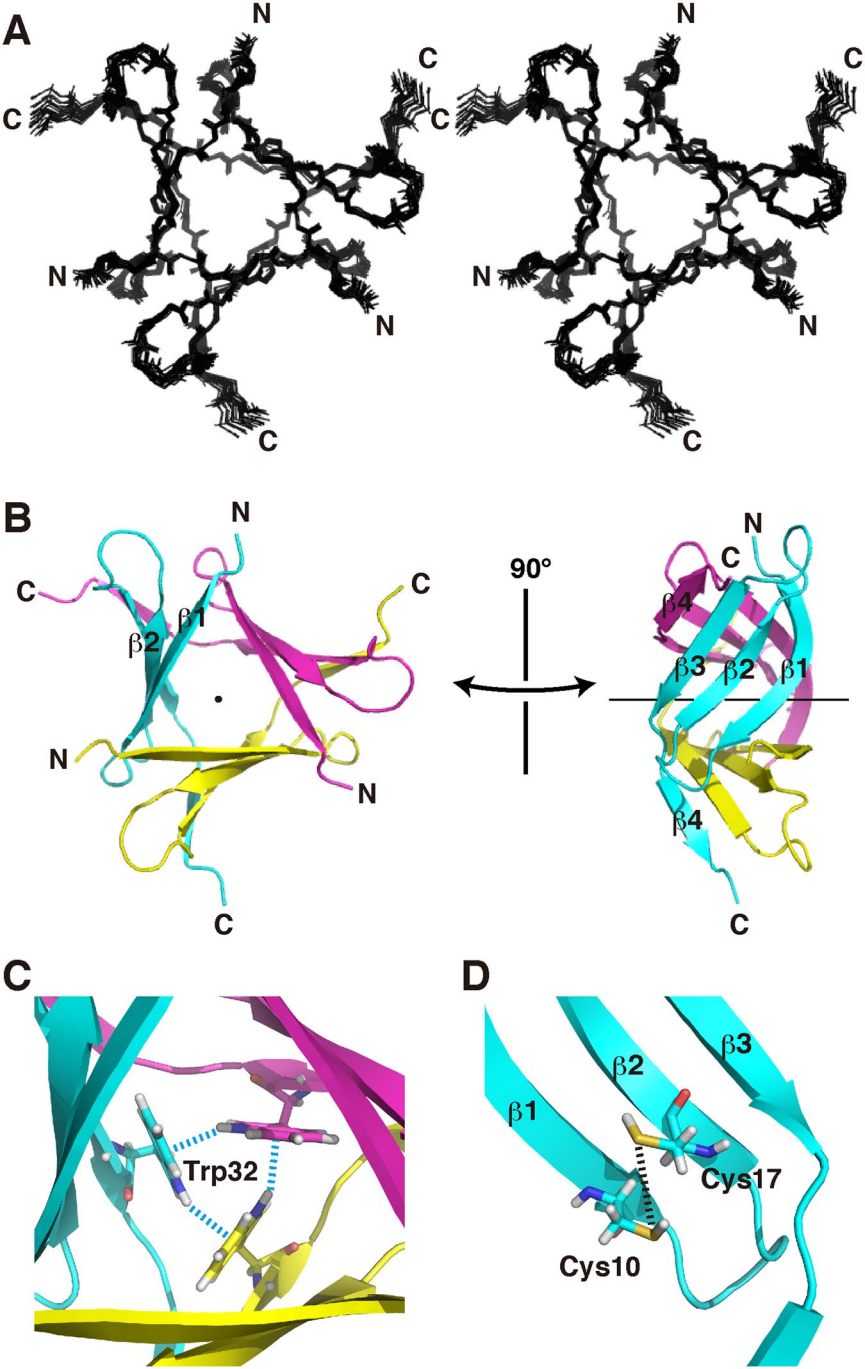
The solution structure of the PhoSL trimer. (**A**) The ensemble of the 20 selected structures in a stereo view. (**B**) Ribbon diagrams of the minimized mean structure where the orientation in the left panel is the same as that in (A). The three monomers are distinguished by different colors. The axis of the rotational symmetry is shown by a dot in left or a horizontal line in right. (**C**) A triad of Trp32 in the structural core. Blue broken lines indicate the NH–π electrostatic interactions. (**D**) Two Cys residues in the vicinity. They should form a disulfide bridge under an oxidized condition, as shown by a broken line. These figures were produced by PyMOL ver. 1.8 (Schrödinger, LLC, New York, USA).

The resulting structure satisfied the structural constraints, i.e., NOE distance constraints and torsional angle constraints, as we observed low root mean square deviations (RMSDs) from these constraints (Table 1). The structure also possessed ideal stereochemical properties, with small van der Waals energy, low RMSDs from ideal geometries, and favorable distributions in the Ramachandran plot. Consequently, it showed good convergence, with low RMSD from the mean structure (Table 1, Fig. 3A).

This solution structure revealed a β-prism scaffold with a clear three-fold rotational symmetry (Fig. 3B). It consists of three β-sheets each containing essentially the four β-strands. The N-terminal and C-terminal halves of β-strand 1, with a kink between Thr6 and Lys7, from different monomers, respectively, form contacts within a short range, from which we may say that the β-sheets expand to five-stranded (Fig. 2C). Also, contacts between β-strands 3 and 4 are intermolecular. Thus, the trimer is tightly connected by swapping of β-strands among the three β-sheets.

At the symmetric center, the indole rings of the three Trp32 contact one another and form the major part of the structural core (Fig. 3C). The contacts are achieved through the edge of a ring plane located over another ring plane, defining relative angle of 120 degrees. The indole Hε1 proton is closest to the ring plane of another Trp with a distance of ~3 Å. It appears, therefore, that there are the NH–π electrostatic interactions, which was considered to be a kind of hydrogen bonds, between the rings^11^. We should point out that in these mutual interactions polarization of the N–H bond itself enhances the density of the π-electrons of the acceptor ring, through electron delocalization. Thus, these interactions should significantly contribute to stabilizing the formation of the structural core. This geometry is consistent with an extremely upfield shifted resonance of the Hε1 proton (4.69 ppm) and NOEs with other ring protons (Fig. S1A in the Supplementary information); a strong ring-current effect^10^ may have overcome the downfield shifting by hydrogen bonding.

Cys10 and Cys17 are located on β-strands 1 and 2, respectively, which are adjacent to each other (Fig. 3D). Because of the reductive condition during the NMR measurements, we observed Hγ protons of these Cys residues and NOEs with other residues (Fig. S1B). Considering their relative positions, however, we can expect a disulfide bridge under an oxidative condition, which is likely to contribute to the structural stability.

### Innate PhoSL structure

Although the PhoSL peptide isolated from the mushroom is 40 amino acids in length^3^, the relevant gene cloned from the mushroom codes for a polypeptide of 180 amino acids^12^ (Fig. S2A). This polypeptide contains three repeats of PhoSL-like sequence, the first of which corresponds to that used in the present study. It is predictable that, at least immediately after the synthesis, the PhoSL of a single polypeptide forms a pseudotrimer. Two homology models of the pseudotrimer were produced on the basis of the present PhoSL trimer structure by SWISS-MODEL system^13^ (Fig. S2B). The two are different in the relative positions of the three repeats in the trimeric structure; either is geometrically allowed by the substantial length of the linkers between the repeats.

Presumably by proteolytic cleavage of the linkers in the mushroom cells, PhoSL becomes a heterogenic trimer of 40 amino acids polypeptides. It is noticeable that the sequence of PhoSL determined by the N-terminal sequence analysis does not exactly match any of the three repeats in the polypeptide (Fig. S2A). On the other hand, respective amino acids of the former match those of either one of the repeats, except for position 27. This suggests a possibility that the 40-amino acid PhoSL peptide from the mushroom is a mixture of those corresponding to the three repeats in the 180-amino acid polypeptide.

It should be noted that the residues that stabilize the structure, as described above, and those that involved in the fucose binding (see below) are conserved among the three repeats (a conservative substitution of Tyr by Phe at position 23 is observed in repeat 2; Fig. S2A). Therefore, the innate PhoSL or the heterogenic trimer probably existing in the cell should be stable and fully functional.

Recently, a core fucose-specific lectin from a bacterium *Streptomyces rapamycinicus* (SL2–1) was identified, which was highly homologous to PhoSL^14^. Interestingly, SL2–1 is ~180 amino acids in length and has three repeats of PhoSL-homologous regions. Therefore, it is likely that the tertiary structure of SL2-1 should be very similar to that of the innate PhoSL protein shown in Fig. S2B. PhoSL, SL2-1, and related proteins from several bacteria and fungi cited in ref.^14^, including another core fucose-specific lectin from *Rhizopus stolonifer*^15^, should comprise a novel family of lectins, which are specific to core fucosylation.

### NMR analysis of binding of fucose

**Figure 4:**
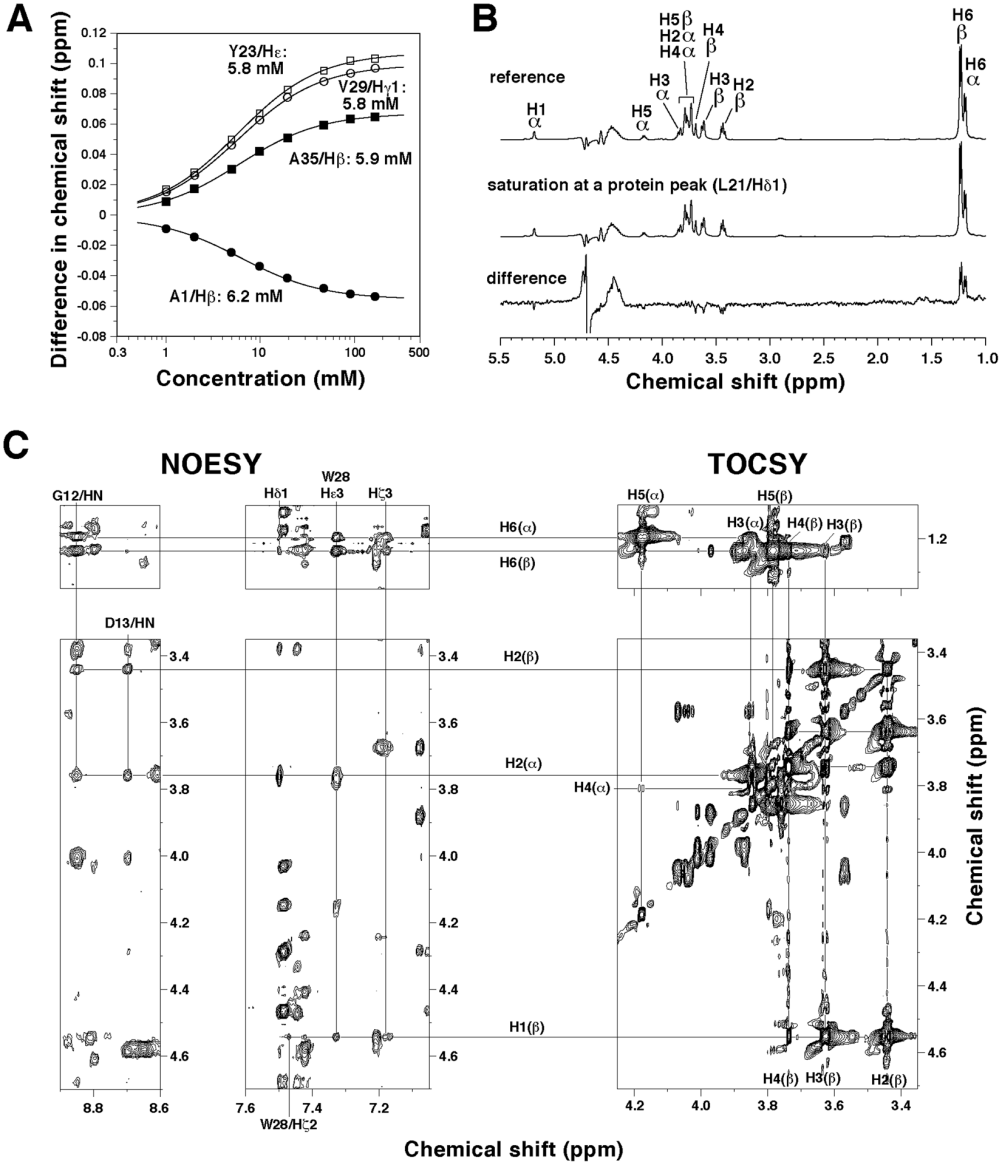
NMR analyses of binding of fucose. (**A**) Changes in chemical shifts for selected resonances of PhoSL upon titration of fucose (see also Fig. S3A). By theoretical fitting of data, *K*_D_ values were obtained, as indicated. (**B**) STD experiment for the interaction of fucose with PhoSL. The solution contained 50 μM PhoSL peptide and 5 mM L-fucose. (**C**) Two-dimensional NMR spectra of PhoSL–fucose mixture solution, containing 0.3 mM PhoSL peptide and 100 mM fucose. (Left four panels) A NOESY spectrum containing NOE cross peaks between PhoSL and the two anomers of fucose. (Right two panels) Those of a TOCSY spectrum showing intramolecular connectivity in the two fucose anomers (right). The mixing times for NOESY and TOCSY were 100 ms and 50 ms, respectively. The NMR assignments for the two anomers appear in literature^30^, and were confirmed here by the 2D NMR. The spectra were recorded at 308 K and at a frequency of 500 MHz in (B) or 900 MHz in (C).

To understand the mechanism of molecular recognition, we examined binding of L-fucose by NMR (Figs 4A and S3A). Upon titration of fucose, chemical shift perturbations, i.e., changes in positions of some PhoSL peaks, were observed. These gradual changes indicate that binding and release of fucose from the protein are fast enough to average the peaks in the free and bound states. Therefore, changes in the chemical shifts reflect those in the relative population. Theoretical fitting for the concentration dependencies yielded dissociation constants (*K*_D_) of 5.8–6.2 mM (5.9 ± 0.2 mM; Fig. 4A). Note that this affinity is weaker than that between PhoSL and N-glycans (~3 μM)^3^ by ~2000-fold. Nonetheless, this difference in *K*_D_s corresponds to that in free energy changes of 4.5 kcal/mol, for which one or two hydrogen bonds may compensate. We suggest, therefore, that there should be such favorable contacts between PhoSL and the other parts of the N-glycans, e.g., GlcNAc moieties, and that the above *K*_D_ value for the fucose monosaccharide is reasonable. This is indeed likely, because an NMR analysis on another core α1–6 fucosylation-specific lectin from an alga *Bryothamnion triquetrum* showed that the 2nd GlcNAc of the N-glycan core is also involved in the binding, in addition to fucose^16^.

L-fucose is a mixture of the two anomers, i.e., α- and β-forms, where the former is biologically relevant. Populations of the α and β anomers were ~30% and ~70%, respectively, as estimated from the NMR peak intensities of H6 methyl protons (Fig. 4B, upper spectrum). Saturation transfer difference (STD) experiment, where saturation of resonances of protein was propagated to those of fucose in rapid equilibrium of association/dissociation, indicated that both the two anomers bound to PhoSL (Fig. 4B). The populations of the anomers bound to PhoSL changed to ~36% and ~64%, as seen in the difference spectra, where the α anomer is likely to be slightly preferable. Thus, we should note that the *K*_D_ value for fucose is an average value for the two anomers in such populations. The STD experiment also showed that H6 methyl group was more involved in the protein binding than the other protons; the only positive peaks in the difference spectrum were those for the H6 protons.

We then analyzed the NOESY spectrum of PhoSL with an excess amount of fucose (100 mM), and compared the chemical shifts of all protons with those of free PhoSL (Fig. S3B). Because chemical shift perturbations are related to changes in microscopic environment, we may predict the residues involved in the fucose binding. The results suggested that regions around the junctions of β-sheet edges were involved.

### Mechanism of molecular recognition

**Figure 5:**
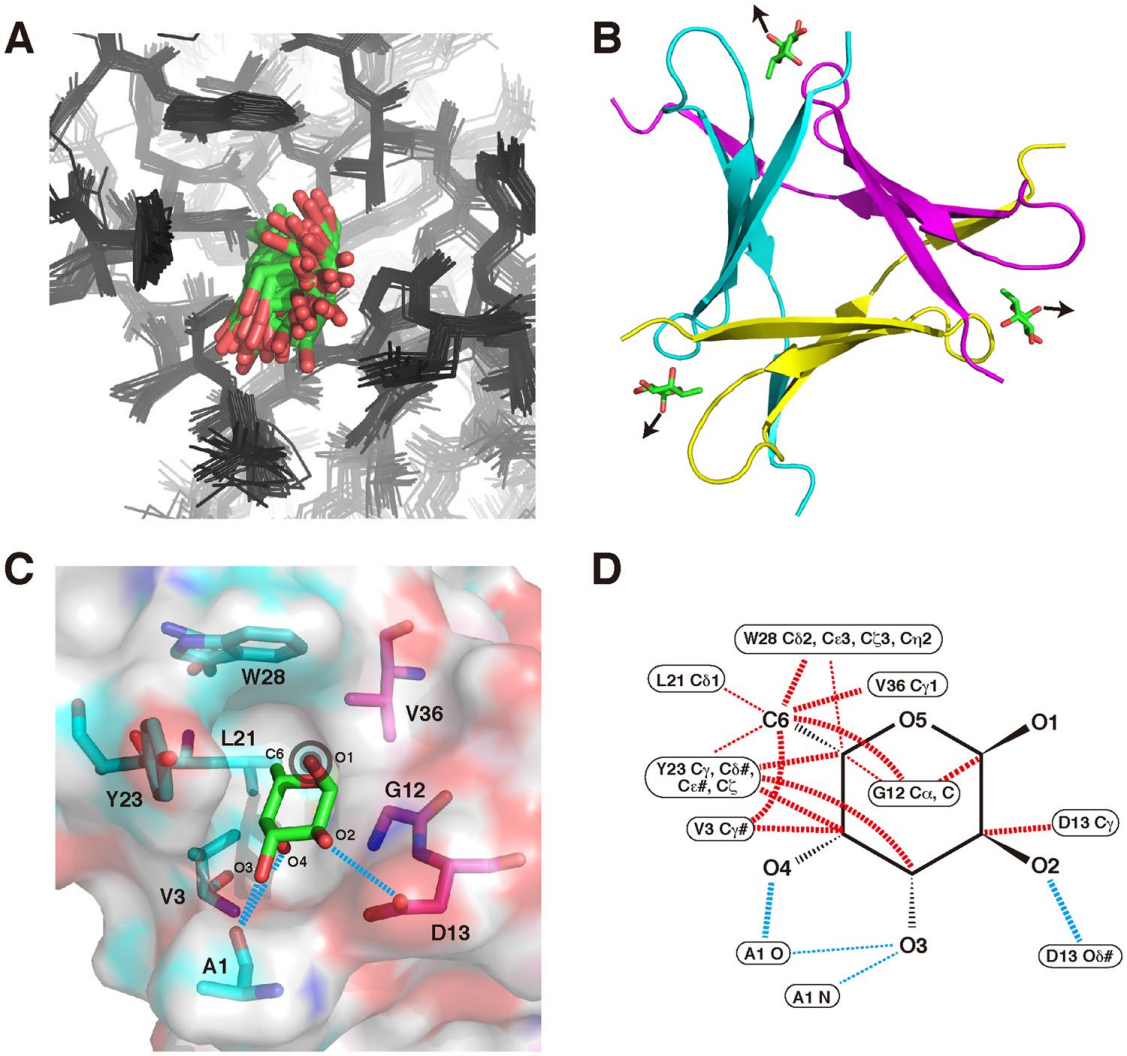
Mechanism for recognition of α-fucose. (**A**) Ensemble of 20 structures of PhoSL (black wire)–α-fucose (stick; green: carbon; red: oxygen) complex obtained by calculations using NOEs between PhoSL and fucose (see Fig. 4C; Table S1). Non-proton atoms in fucose and surrounding residues (Ala1, Val3, Gly12, Asp13, Leu21, Tyr23, Trp28, and Val36) are aligned. (**B**) The minimized mean structure of PhoSL (ribbon)–α-fucose (stick) complex, where the PhoSL monomers are shown in different colors. Arrows indicate presumable directions of the α1–6 linkage to the GlcNAc of the N-glycan. (**C**) A binding pocket for α-fucose seen in the surface of the minimized mean structure. The residues that comprise the pocket are indicated in stick, as colored for the protein chains. Fucose–protein hydrogen bonds (distances ≤3.5 Å between N or O atoms) are shown by blue broken lines. As in (B), a sign on the O_1_ atom indicates the direction of α1–6 linkage to GlcNAc, pointing toward this side of the plane. (**D**) Schematic representations of contacts between PhoSL and α-fucose. Hydrophobic contacts (distances ≤4.5 Å between C atoms: red) and hydrogen bonds (defined above: blue) are indicated by broken lines. Bold lines indicate the contacts that are observed in 50% or more of the 60 pockets in the structure ensemble, while thin lines indicate those that are observed in 20% or more. The figures in (A–C) were produced by PyMOL.

In the same NOESY spectrum, we identified several NOE cross-peaks between PhoSL and fucose, although the molecules are in an equilibrium state, with rapid binding and release (Fig. 4C). We separately assigned the cross-peaks for the two anomers of fucose, and calculated the respective structures in a similar manner for the free PhoSL (Figs 5 and S4; Table S1). With these NOEs, the structure around the bound fucose did not strongly converge (Figs 5A and S4A). Nonetheless, the binding site and orientation became clear in the structures. The following descriptions on the binding mechanism are for α-fucose, although they are essentially the same for β-fucose, unless otherwise stated.

The fucose-binding sites were located indeed where the chemical shift perturbation suggested, i.e., junctions of the β-sheet edges (Fig. 5B). At each site, a pocket was formed by several residues from two PhoSL chains (Fig. 5C). Fucose molecule deeply entered the pocket, with the C6 methyl group placed at the bottom, which was consistent with the STD result. Between fucose and the residues that comprise the pocket, a number of hydrophobic and hydrogen-bonding interactions are formed (Fig. 5C,D). Namely, the C6 methyl group and other carbon atoms contact aliphatic side chains of Val3, Leu21, and Val36, aromatic side chains of Tyr23 and Trp28, backbone carbon of Gly12, and aliphatic moiety of Asp13. Of noticeable is the contacts to Y23, which have a CH–π attractive effect^17^, other than the hydrophobic effect. Also, hydrogen bonds are formed between the O_2_, O_3_, or O_4_ hydroxyl groups and nitrogen or oxygen atoms of Ala1, and Asp13 (Gly12 is also involved in hydrogen bonds for β-fucose; Fig. S4C,D). Because fucose in the pocket is not strongly fixed in the structural ensemble, some of these contacts are formed in a partial fraction; this may be more fixed when GlcNAc or N-glycan is attached. It should be noted that the O_1_ atom of α-fucose points outward from the pocket and is open for connection to GlcNAc (Fig. 5B,C).

In the present study, we identified the binding site of fucose and a number of intermolecular interactions, which provided the structural basis for the molecular recognition. To reveal the mechanism of specificity for the α1–6 linkage, however, it will be necessary to analyze the structure of complex with a sugar containing GlcNAc. There may be favorable contacts between GlcNAc and PhoSL, or machinery for discrimination against the other types of linkages.

### Sharing four-stranded antiparallel β-sheets with other lectins

**Figure 6:**
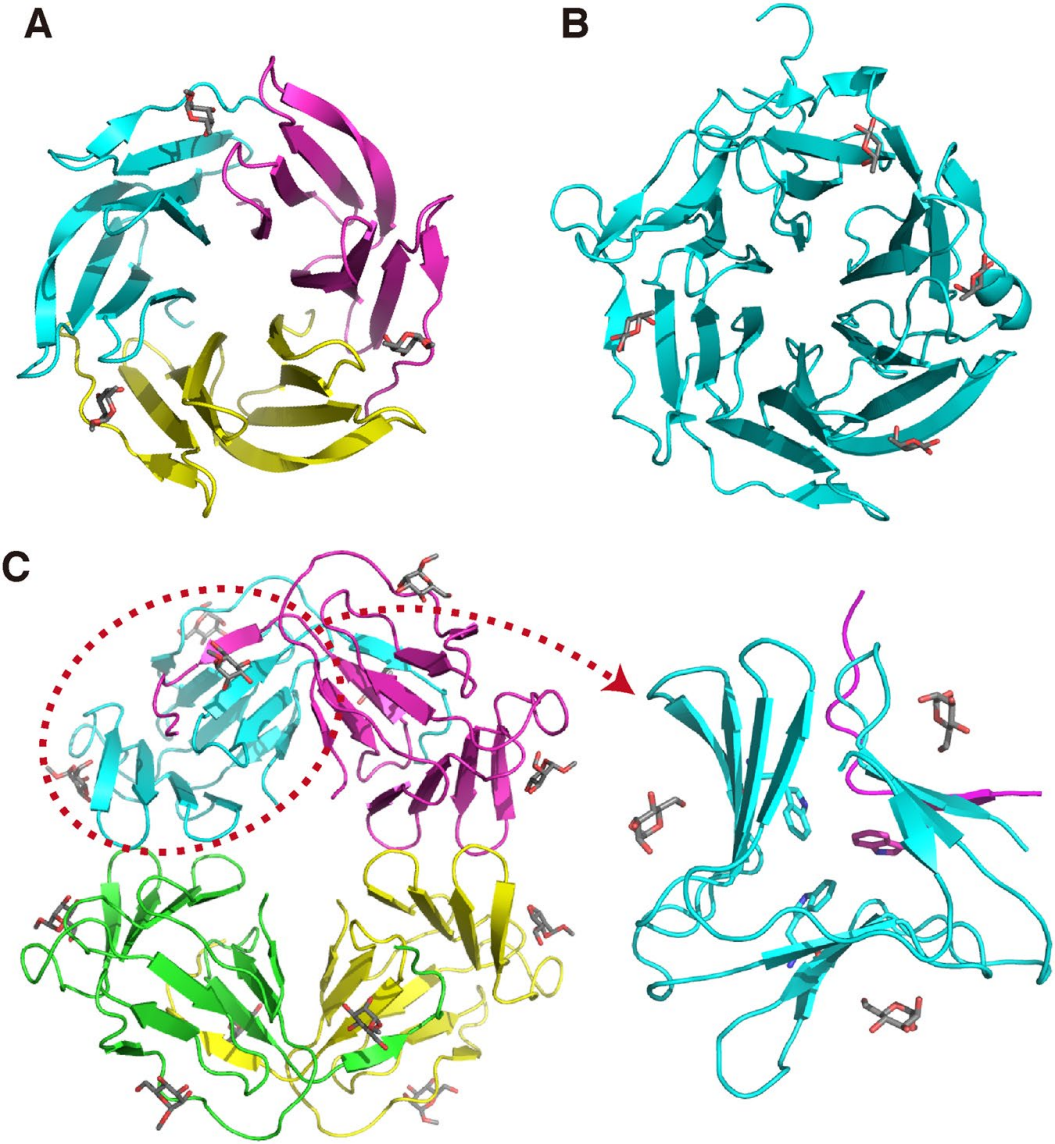
Comparison of related lectin structures. (**A**) The crystal structure of RSL trimer in complex with methylfucose (PDB ID: 2BT9)^18^. (**B**) The crystal structure of AAL, consisting of a single polypeptide, in complex with fucose (PDB ID: 1OFZ)^19^. (**C**) The crystal structure of GNA tetramer in complex with methylmannose (PDB ID: 1MSA)^20^. In the right panel, a monomeric part of GNA with a swapping of a β-strand (circled in the left panel) is focused. In these figures, different polypeptide chains are distinguished by colors, and bound sugars are shown in stick representations. In the right panel of (C), a triad of Trp residues in the core is indicated also by stick.

A fucose-binding lectin from a bacterium *Ralstonia solanecearum* (RSL) forms a symmetric trimer as well (Fig. 6A)^18^. A monomer consists of a tandem of two four-stranded antiparallel β-sheets, resulting in a six-bladed β-propeller structure with a pseudo-six-fold symmetry for the trimer.

A very similar six-bladed β-propeller structure is observed for another fucose-binding lectin from a fungus *Aleuria aurantia* (AAL) (Fig. 6B)^19^. The latter, however, is a monomeric polypeptide possessing six repeats of four-stranded β-sheets.

A mannose-specific lectin (or agglutinin) from a higher plant *Galanthus nivalis* (GNA) forms a tetramer (Fig. 6C)^20^. Each monomer consists of three four-stranded antiparallel β-sheets. When two of the three β-sheets in each monomer are considered, the structure of the tetramer appears to be an eight-bladed β-propeller structure partly similar to those of RSL and AAL. On the other hand, the three β-sheets in the monomeric part are arranged in a symmetric manner and comprise a pseudotrimeric β-prism structure similar to that of PhoSL (Fig. 6C, right panel). It also contains a triad of Trp residues at this pseudosymmetric center.

Nonetheless, the above resemblance of GNA monomer to PhoSL trimer is superficial. Namely, β-prism of GNA is formed essentially by a single polypeptide, with swapping of one β-strand with another monomer. Also, the β-strands in the front layer direct clockwise, while the corresponding β-strands of PhoSL direct counterclockwise (Figs 2B and 6C); this is also true when the structures are looked from the opposite direction. In addition the three Trp rings of GNA contact among one another through simple hydrophobic forces, but not through the NH–π electrostatic forces. Furthermore, the binding pockets for sugar are formed over the β-sheet planes.

As above, RSL, AAL, and GNA commonly contain four-stranded antiparallel β-sheets as units. By rearrangements in the polypeptide level, they form β-propellers or similar structures. PhoSL is similar to them only in that it also contains similar antiparallel β-sheet as the structural unit. Indeed, when we used the Dali program^21^ to search the data base for similar structures, only those possessing antiparallel β-sheets of similar length, including several β-propeller structures, were identified. This was also essentially the same when we artificially treated the PhoSL trimer as a single polypeptide before submitting to Dali. Therefore, the arrangement of the three β-sheets in PhoSL defines a novel type of structural scaffold.

## Materials and Methods

### Sample preparation

A PhoSL peptide without N-terminal acetylation or C-terminal amidation (APVPVTKLVCDGDTYKCTAYLDYGDGKWVAQWDTAVFHTT) was chemically synthesized by the standard solid-phase method and purified to >95% by HPLC (BEX Co., Ltd., Tokyo, Japan). The above sequence is derived from the gene relevant to the lectin described in a published patent application^12^ and is different from that in the literature^3^, APVPVTKLVCDGDTYKCTAYLDFGDGRWVAQWDTNVFHTG, at four positions (23rd, 27th, 35th, and 40th amino acids from the N-terminus, as underlined; see Fig. S2A). It was shown, however, that these alterations do not influence the specificity to core-fucosylated glycans^12^. In this patent, it was mistakenly described that this lectin was derived from a mushroom *P. terrestris*. However, it was later corrected to be derived from *P. squarrosa* in literature^3^. The concentration of the peptide in solutions was estimated from absorbance at 280 nm (A_280_), where molar absorption coefficient was calculated from the amino acid sequences^22^. The molar concentrations for PhoSL are shown as a monomer, unless otherwise stated.

### Gel filtration analysis

The PhoSL peptide (96 μM: 0.43 mg mL^−1^) and molecular marker proteins (0.5 mg mL^−1^), i.e., bovine serum albumin (69.3 kDa; Sigma-Aldrich), ovalbumin (42.9 kDa; Sigma-Aldrich, carbonic anhydrase (29.0 kDa; Sigma-Aldrich), and ribonuclease A (13.7 kDa; Sigma-Aldrich), were dissolved in 20 mM Tris-HCl (pH 7.5), 100 mM NaCl. The solutions of 200 μL each were analyzed separately on a Superdex 75 10/300 column (GE Healthcare).

### Cross-linking analysis

PhoSL (0.2 mM) was dissolved in 20 mM 4-(2-hydroxyethyl)-1-piperazine-ethanesulfonic acid (HEPES) (pH 7.5) (DOJIN, Kumamoto, Japan), 50 mM EDC (Peptide Institute, Inc., Osaka, Japan), 50 mM NHS (Wako, Osaka, Japan), and kept at room temperature for 1 h, allowing the linking reaction. This was stopped by adding sample solution for SDS-PAGE [50 mM Tris-HCl (pH 6.8), 2% SDS, 6% β-mercaptoethanol, 10% glycerol, and 0.02% bromophenol blue: final concentrations] and heating at 90 °C for 5 min. After SDS-PAGE, gel moieties containing bands were cut out. The gels were incubated in 100 mM NH_4_HCO_3_ for 10 min and then crushed with a homogenization pestle. After purified with ZipTip C4 (Millipore) according to the protocol provided by the vendor, proteins were analyzed by mass spectrometry on Shimadzu Axima TOF^2^.

### NMR measurements

For structure determination of PhoSL trimer, solution consisted of 0.3 mM PhoSL peptide, 20 mM d_11_-Tris-HCl (pH 7.5) (Isotec Inc.), 1 mM d_10_-dithiothreitol (DTT) (Isotec Inc.), 0.1 mM sodium 2,2-dimethyl-2-silapentane-5-sulfonate (DSS), and 5% D_2_O. In PhoSL-fucose titration analysis, a solution containing 50 μM PhoSL peptide, 20 mM d_11_-Tris-HCl (pH 7.5), 0.2 mM d_10_-DTT, 0.1 mM DSS, and 5% D_2_O was titrated with a solution of 500 mM or 1M L-fucose (Tokyo Chemical Industry, Co., Ltd., Tokyo, Japan) in 20 mM d_11_-Tris-HCl (pH 7.5). Solution for STD^23^ experiment consisted of 50 μM PhoSL peptide, 5 mM L-fucose, 20 mM d_11_-Tris-HCl (pH 7.5), 0.2 mM d_10_-DTT, 0.1 mM DSS, and 5% D_2_O. That for structure determination of PhoSL–fucose complex or analysis of chemical shift perturbation consisted of 0.3 mM PhoSL peptide, 100 mM L-fucose, 20 mM d_11_-Tris-HCl (pH 7.5), 1 mM d_10_-DTT, 0.1 mM DSS, and 5% D_2_O.

NOE spectroscopy (NOESY), total correlation spectroscopy (TOCSY), and double-quantum-filtered correlation spectroscopy (DQF-COSY) spectra^10^ for structure determination and chemical shift perturbation were recorded on Bruker Avance III-900 (900.13 MHz for ^1^H) spectrometers at 308 K, where mixing times of 100 ms and 50 ms for NOESY and TOCSY, respectively. Fucose-titration and STD experiments were performed on a Bruker Avance III-500 (500.13 MHz for ^1^H) spectrometer at 308 K. For STD, a spectrum with irradiation on resonance of Leu21/H_δ_1 for 2 sec was subtracted from a reference spectrum with an off-resonance irradiation. NOESY, TOCSY, and simple ^1^H spectra of a sample dissolved in 99.96% D_2_O (Isotec Inc.) after lyophilization were recorded at 298 K, where 20 hydrogen bond donors were identified.

### Spectral analysis and structure determination of PhoSL trimer

The NOESY, TOCSY, and DQF-COSY spectra were analyzed by Felix ver. 2007 (Felix NMR, Inc., San Diego, CA). The backbone and side chain ^1^H resonances were assigned on the basis of the sequential NOEs^10^. Chemical shifts were referenced to the internal DSS. By analyzing the spectra, 14 and 5 pairs of H_β_ and valine H_γ_ resonances, respectively, were assigned stereospecifically and, at the same time, the relevant rotamers around the χ^1^ angles were determined (methods described in refs^24,25^). Also, two pairs of Leu H_δ_ resonances were spectrospecifically assigned, and the relevant χ^2^ rotamers were determined. Similarly, the χ^1^ rotamers of three Thr residues were estimated.

A simulated annealing protocol with random initial velocities^26^ and an extended initial structure was carried out by CNS ver. 1.3^27^. The distance constraints derived from the NOESY spectra and torsion angle constrains on the determined χ^1^ and χ^2^ rotamers were imposed as described previously^28^. For structure determination of the symmetric trimer, weak non-crystallographic symmetry (NCS) restraints were applied at 10 kcal mol^−1^ Å^−2^. Conditions for the molecular dynamics were: 50,000 K for 2,000 steps in torsion-angle space at high-temperature annealing stage; 50,000 K (initial) for 2,000 steps in torsion-angle space at the first slow cooling stage; and 2,000 K (initial) for 2,000 steps in Cartesian space at the second slow cooling stage.

NOEs were categorized with regard to inter- or intramolecular ones. All the sequential NOEs (NOEs between adjacent residues) were treated as intramolecular ones. For medium-range (2 ≤ |i–j| ≤ 4; i and j are residue numbers of the two protons for the NOE) and long-range (|i–j| > 4) NOEs, ambiguity in terms of this classification was introduced by means of the sum-averaging function. In order to reduce the search space of the random simulated annealing and increase the acceptance ratio, however, some NOEs from the interstrand interfaces were fixed as either intramolecular or intermolecular ones. This was systematically introduced, as follows.

When NOEs around the interface between β-strands 3 and 4, i.e., those between regions Lys27–Gln31 and Thr34–Thr39, which included typical interstrand backbone NOEs (Val29/HN–Phe37/HN, Ala30/Hα–Val36/Hα, and Gln31/HN–Ala35/HN in Fig. 2A), were treated as intramolecular ones, we could not obtain any acceptable structures on the basis without a distance violation greater than 0.2 Å, no torsion angle violation greater than 2° from 3000 trials. Alternatively, when these NOEs were treated as intermolecular ones, we obtained eight acceptable structures from 3000 trials. In all of the accepted structures, the strands were arranged as shown in Fig. 2C. Therefore, NOEs around other interfaces between β-strands were fixed accordingly. Namely, the NOEs were intermolecular for Pro2–Val5 and Val9–Gly12 (the N-terminal and C-terminal halves of β-strand 1); intramolecular for Val3–Val9 and Cys17–Tyr23 (β-strands 1 and 2); and intramolecular for Lys16–Leu21 and Lys27–Thr34 (β-strands 2 and 3). Based on the calculated structure, acceptors of the hydrogen bonds were determined, and the relevant distance constraints were applied. Finally, in 50 trials, 20 structures were selected on the bases without a distance violation greater than 0.2 Å, no torsion angle violation greater than 2°, and the lowest total energies.

The structures of PhoSL–α-fucose and PhoSL–β-fucose complexes were calculated respectively by a simulated annealing protocol similar to the above. Intensities of the NOEs between PhoSL and either of the two anomers were calibrated to the relative occupancies seen in the STD experiment (Fig. 4B), as if the binding pocket was fully occupied by a particular anomer. For the initial structure of the calculation, the minimized mean structure obtained by a calculation in advance without the PhoSL–fucose NOEs was used, instead of the extended structure. Conditions for the molecular dynamics were weakened, i.e., 1,000 K for 1,000 steps in torsion-angle space at the annealing stage; 1,000 K (initial) for 1,000 steps in torsion-angle space at the first cooling stage; and 1,000 K (initial) for 1,000 steps in Cartesian space at the second cooling stage. The NCS restraints were applied only to the protein moiety. Calculation was performed for 20 trials, and all of the structures were accepted, without a distance violation greater than 0.2 Å or a torsion angle violation greater than 2°.

### Accession numbers

The co-ordinates of the determined structure and ^1^H chemical shifts for the free state of PhoSL have been deposited to the Protein Data Bank (PDB) and BMRB under accession IDs 5xzk and 36103, respectively.

## Electronic supplementary material

Supplementary Information
