# Supplementary material for: The trimeric solution structure and fucose-binding mechanism of the core fucosylation-specific lectin PhoSL
Source: Sci Rep. 2018 May 17;8:7740. doi: 10.1038/s41598-018-25630-2 (PMC5958098; doi:10.1038/s41598-018-25630-2)
Supplement: Supplementary file 1 — Supplementary Information [file 41598_2018_25630_MOESM1_ESM.pdf]

Supplementary Information

The trimeric solution structure and fucose-binding mechanism of the core fucosylation-specific lectin PhoSL

Kazuhiko Yamasaki[1,*], Tomoko Yamasaki[1], and Hiroaki Tateno[2]

From [1]Biomedical Research Institute, and [2]Biotechnology Research Institute for Drug Discovery, National Institute of Advanced Industrial Science and Technology (AIST), Tsukuba [1]305-8566 and [2]305-8568, Japan.

Table of Contents

Supplementary Table

Table S1: Structural statistics for PhoSL-fucose complex structures.

Supplementary Figures

Figure S1: Resonances of Trp32 Hε1 and Cys10/Cys17 Hγ protons.

Figure S2: Innate PhoSL protein.

Figure S3: Chemical shift perturbation upon titration of fucose.

Figure S4: Mechanism for recognition of β-fucose.

Supplementary table

Table S1. Structural statistics for the NMR structures of PhoSL trimer–fucose complexes

	α-fucose	β-fucose
Structural constraints (per trimer)		
PhoSL-fucose NOE constraints[a]	48	63
NOE constraints within PhoSL[a]	1662	1662
Hydrogen bonds in PhoSL	120	120
Torsion angles in PhoSL	81	81
Total	1911	1926
Characteristics	Ensemble of 20 structures (minimized mean structure)	
R.m.s. deviation from constraints		
NOEs (Å)	0.0040 ± 0.0009 (0.03)	0.0036 ± 0.0005 (0.03)
Torsion angles (degrees)	0.000 ± 0.000 (0.000)	0.003 ± 0.008 (0.000)
Noncrystallographic symmetry (kcal/mol)	0.01 ± 0.0 (0.003)	0.07 ± 0.03 (0.04)
Van der Waals energy (kcal/mol)[b]	9.6 ± 2.6 (7.1)	9.7 ± 2.4 (8.3)
R.m.s. deviation from the ideal geometry		
Bond lengths (Å)	0.0009± 0.0001 (0.0008)	0.0009 ± 0.0001 (0.0009)
Bond angles (degrees)	0.279 ± 0.005 (0.271)	0.289 ± 0.013 (0.284)
Improper angles (degrees)	0.159 ± 0.006 (0.153)	0.163 ± 0.005 (0.159)

[a]Each NOE corresponds to three constraints for the symmetric trimer.

[b]Values calculated with the repulsive nonbonded energy function in the CNS software package.

Supplementary Figures

Legends

Figure S1: Resonances of (A) Trp32 Hε1 and (B) Cys10/Cys17 Hγ protons (4.69, 1.21, and 1.12 ppm, respectively). Relevant cross peaks in NOESY (black) and TOCSY (red) are indicated. These spectra were recorded at 308 K and at a frequency of 900 MHz. The mixing times for NOESY and TOCSY were 100 ms and 50 ms, respectively.

Figure S2: Innate PhoSL protein. (A) Amino acid sequence encoded by the PhoSL gene (ref. 12 in the main text), which is accompanied by that of the purified PhoSL, as determined by amino acid analysis (ref. 3 in the main text; bottom). The amino acids that are different from those of the purified PhoSL are marked in red. The positions of β-strands are shown as in Fig. 2 in the main text; the peptide used in the present structural analysis corresponds to the first repeat of the protein (Ala1–Thr40). (B) Homology models of the structure of the innate PhoSL protein as produced by SWISS-MODEL (ref. 13 in the main text). Rainbow color from the N-terminus (blue) to the C-terminus (red) is applied. Figure was produced by PyMOL.

Figure S3: Chemical shift perturbation upon titration of fucose. (A) NMR spectra at different concentrations of fucose as shown in left. Selected regions relevant for Fig. 4A in the main text are expanded below. (B) Chemical shift perturbation induced by 100 mM fucose, as analyzed by 2D NMR. Red and yellow spheres in the trimer structure indicate that differences in chemical shifts are ≥0.2 ppm and ≥0.1 ppm, respectively. The junctions of β-sheet edges are shown by arrows.

Figure S4: Mechanism for recognition of β-fucose. (A) Structural ensemble and (B) the minimized mean structure of PhoSL–β-fucose complex. (C) A binding pocket for β-fucose. (D) A summary of contacts between PhoSL and β-fucose. Representation schemes are the same as those in Fig. 5 in the main text.

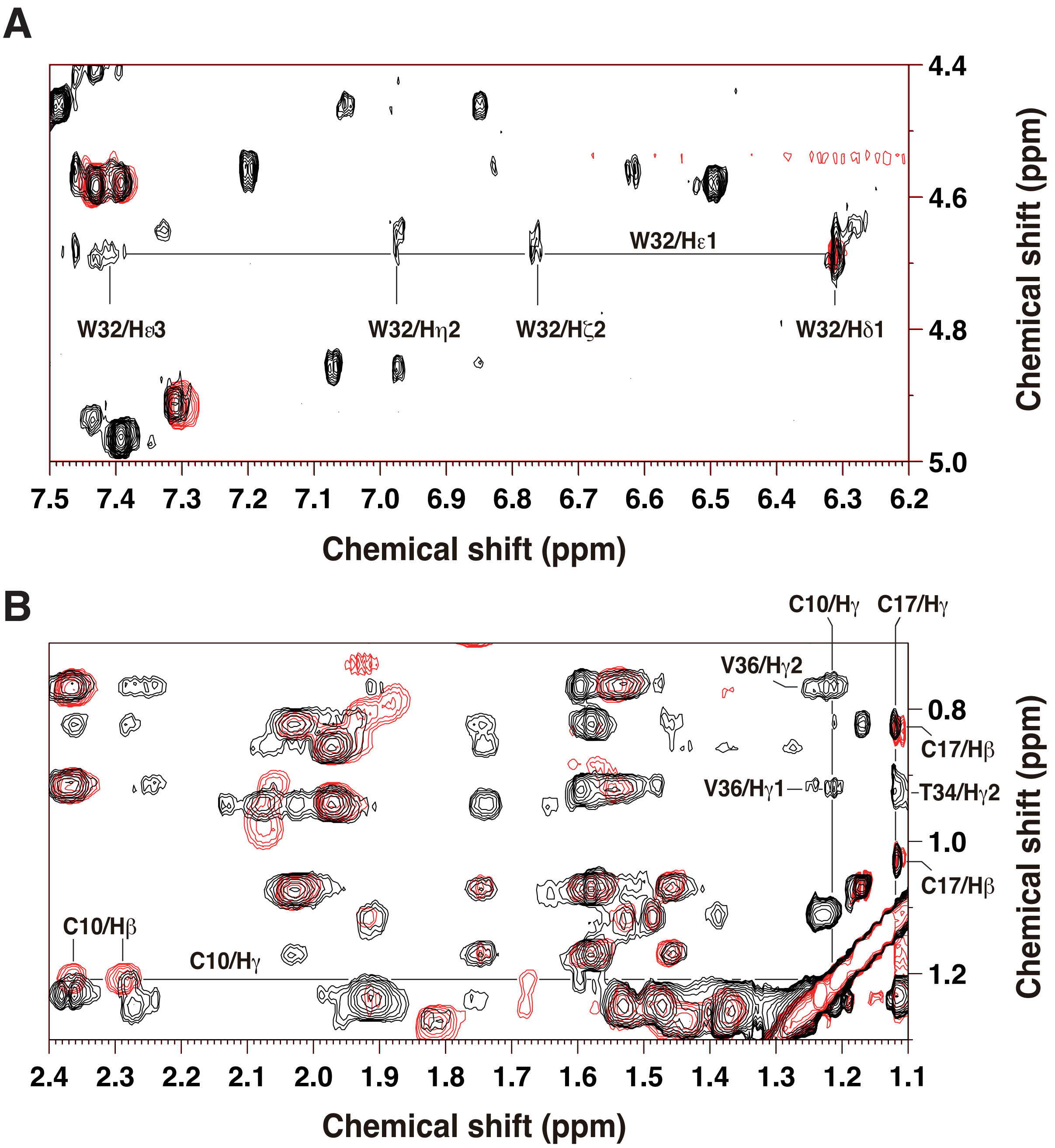

A
W32/Hε1
W32/Hε3
W32/Hη2
W32/Hζ2
W32/Hδ1
4.4
4.6
4.8
5.0
7.5 7.4 7.3 7.2 7.1 7.0 6.9 6.8 6.7 6.6 6.5 6.4 6.3 6.2
Chemical shift (ppm)
Chemical shift (ppm)
B
C10/Hγ
C17/Hγ
V36/Hγ2
C17/Hβ
V36/Hγ1
T34/Hγ2
C17/Hβ
C10/Hβ
C10/Hγ
0.8
1.0
1.2
2.4 2.3 2.2 2.1 2.0 1.9 1.8 1.7 1.6 1.5 1.4 1.3 1.2 1.1
Chemical shift (ppm)
Chemical shift (ppm)

Fig. S1

A

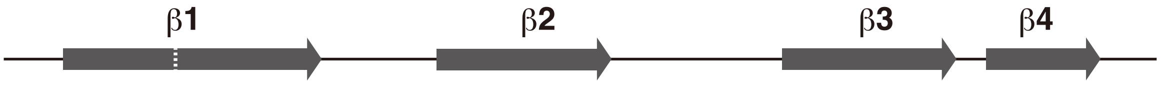


```
          1        10        20        30        40
31aa- APVPVTKLVCDGDTYKCTAYLDYGDGKWVAQWDTAVFHTT
15aa- APVPVSKLVCDGDTYKCTATLDFGDGHWVAQWSANIFHQG
 8aa- APVPVTKLVCDGDTYKCTATLDYGDSNWVAQWGTSVFHTS-6aa

      APVPVTKLVCDGDTYKCTAYLDFGDGRWVAQWDTNVFHTG
```

(PhoSL peptide in Kobayashi *et al.*, 2012: ref. 3)

B

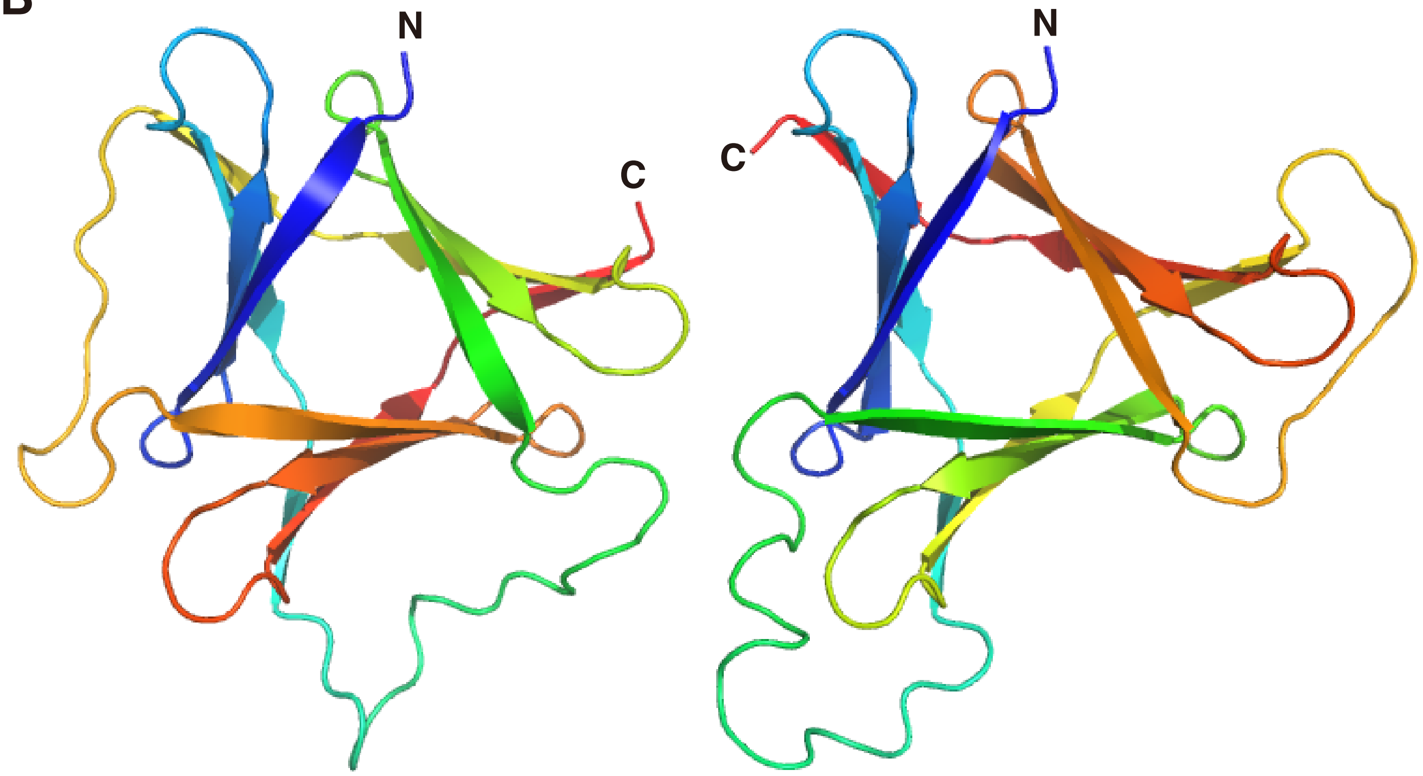

Fig. S2

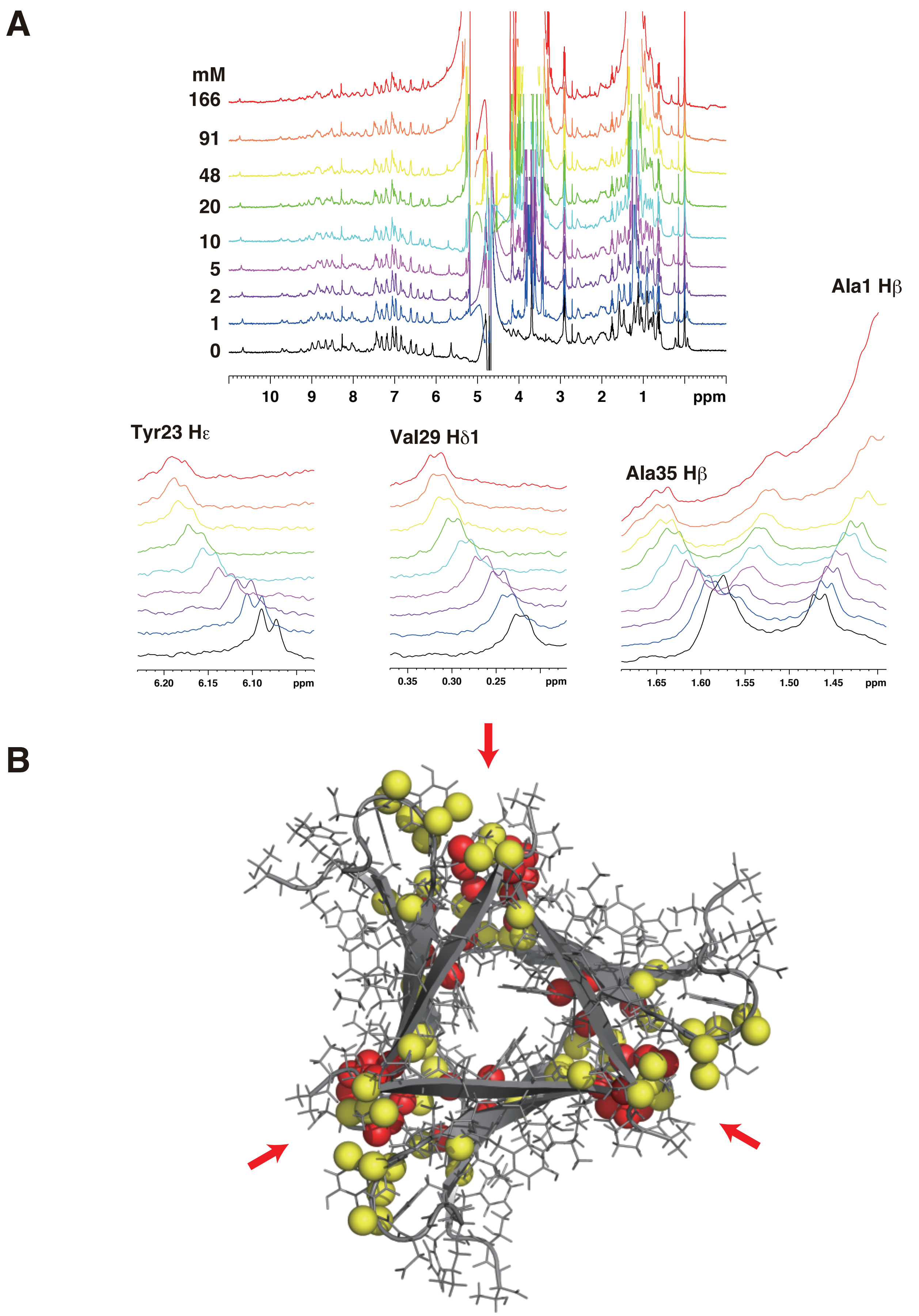
A
mM
166
91
48
20
10
5
2
1
0
10
9
8
7
6
5
4
3
2
1
ppm
Ala1 Hβ
Tyr23 Hε
Val29 Hδ1
Ala35 Hβ
6.20
6.15
6.10
ppm
0.35
0.30
0.25
ppm
1.65
1.60
1.55
1.50
1.45
ppm
B

Fig. S3

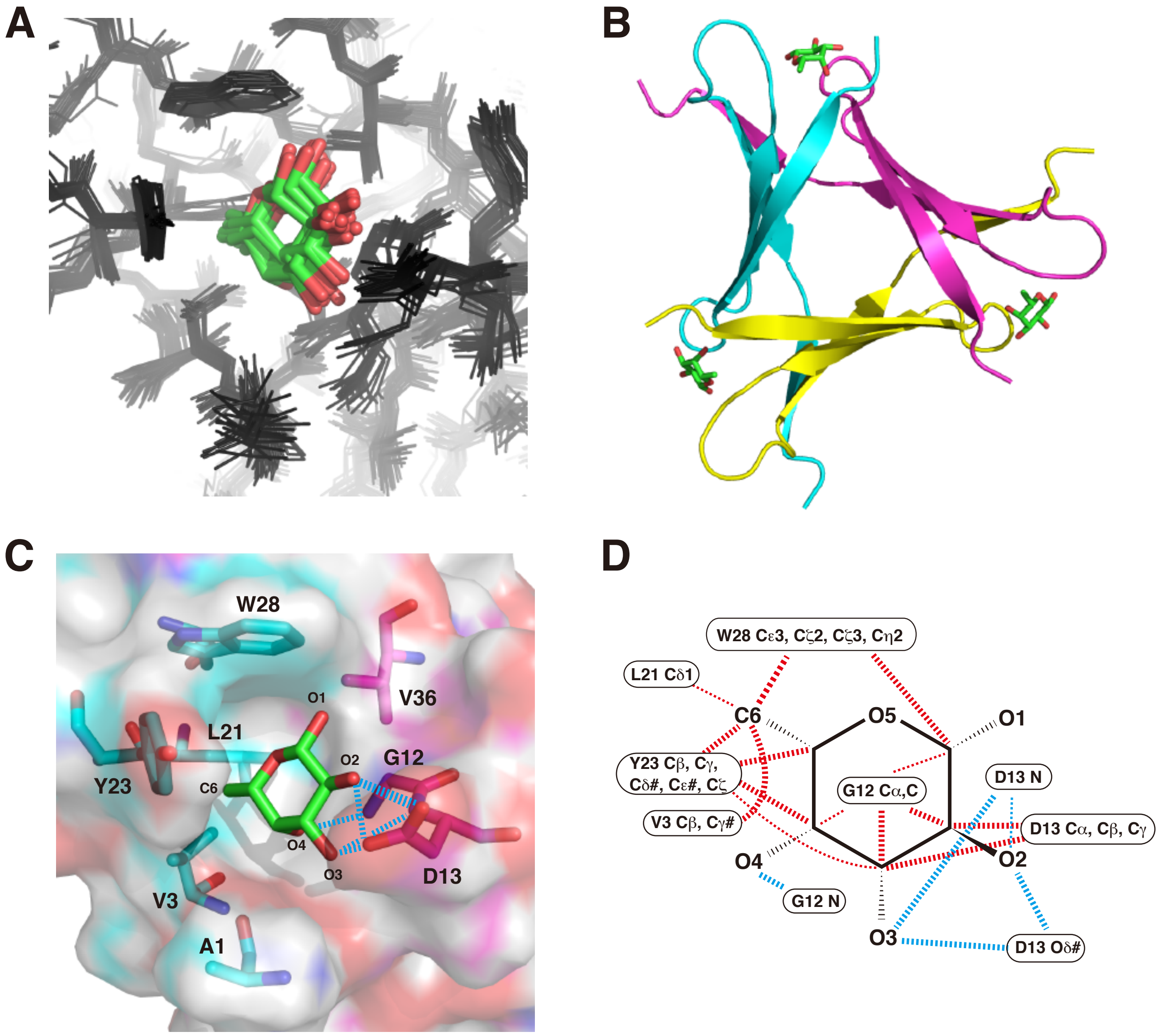
A
B
C
W28
O1
V36
L21
O2
G12
Y23
C6
O4
O3
D13
V3
A1
D
W28 Cε3, Cζ2, Cζ3, Cη2
L21 Cδ1
C6
O5
O1
Y23 Cβ, Cγ, Cδ#, Cε#, Cζ
G12 Cα,C
D13 N
V3 Cβ, Cγ#
D13 Cα, Cβ, Cγ
O4
O2
G12 N
O3
D13 Oδ#

Fig. S4
